# Comparison of Magnetic Resonance Imaging in Live vs. Post Mortem Rat Brains

**DOI:** 10.1371/journal.pone.0071027

**Published:** 2013-08-13

**Authors:** Ipek Oguz, Richard Yaxley, Francois Budin, Marion Hoogstoel, Joohwi Lee, Eric Maltbie, Wen Liu, Fulton T. Crews

**Affiliations:** 1 University of North Carolina at Chapel Hill, Department of Psychiatry, Chapel Hill, North Carolina, United States of America; 2 University of North Carolina at Chapel Hill, Department of Computer Science, Chapel Hill, North Carolina, United States of America; 3 University of North Carolina at Chapel Hill, Bowles Center for Alcohol Studies, Chapel Hill, North Carolina, United States of America; National Institute of Radiological Sciences, Japan

## Abstract

Magnetic Resonance Imaging (MRI) is an increasingly popular technique for examining neurobiology in rodents because it is both noninvasive and nondestructive. MRI scans can be acquired from either live or post mortem specimens. *In vivo* scans have a key advantage in that subjects can be scanned at multiple time-points in longitudinal studies. However, repeated exposure to anesthesia and stress may confound studies. In contrast, post mortem scans offer improved image quality and increased signal-to-noise ratio (SNR) due to several key advantages: First, the images are not disrupted by motion and pulsation artifacts. Second, they allow the brain tissue to be perfused with contrast agents, enhancing tissue contrast. Third, they allow longer image acquisition times, yielding higher resolution and/or improved SNR. Fourth, they allow assessment of groups of animals at the same age without scheduling complications. Despite these advantages, researchers are often skeptical of post mortem MRI scans because of uncertainty about whether the fixation process alters the MRI measurements. To address these concerns, we present a thorough comparative study of *in vivo* and post mortem MRI scans in healthy male Wistar rats at three age points throughout adolescence (postnatal days 28 through 80). For each subject, an *in vivo* scan was acquired, followed by perfusion and two post mortem scans at two different MRI facilities. The goal was to assess robustness of measurements, to detect any changes in volumetric measurements after fixation, and to investigate any differential bias that may exist between image acquisition techniques. We present this volumetric analysis for comparison of 22 anatomical structures between *in vivo* and post mortem scans. No significant changes in volumetric measurements were detected; however, as hypothesized, the image quality is dramatically improved in post mortem scans. These findings illustrate the validity and utility of using post mortem scans in volumetric neurobiological studies.

## Introduction

MRI has proven to be a valuable complement to traditional histology and can serve as a hypothesis generation method for more focused histological and molecular examinations after the scanning has been performed.

One of the major design decisions in rodent MRI experiments is whether to directly scan live animals or to use fixed brains instead. *In vivo* studies have clear advantages in terms of allowing longitudinal study designs, increased flexibility in experimental design for studies that involve behavioral or other similar measurements that require the animal to be live, as well as not risking the occasional failed perfusion. However, *in vivo* scans also have several disadvantages, such as motion and pulsation artifacts in the acquired data, limited scan time (and therefore limited resolution and/or limited signal-to-noise ratio) and stress to the animal. An additional issue is that the anesthesia required for *in vivo* scans, which can be considered a confound in studies of drugs or alcohol and may affect the developmental trajectory in young animals which may potentially bias longitudinal studies. Similarly, restraint stress is a very important concern which can be a confound in studies. In fact, restraint stress in rodents is used to model stress-induced pathology, including changes in hormones, neurotransmitters, neuroimmune activation and degeneration [1]–[6]. A more practical concern is regarding the logistics of scheduling the scans when assessing the brain at specific ages. Most facilities can not scan multiple live animals at the same time, limiting the maximum number of *in vivo* scans per day. This requires staggering of either the dates of birth of the animals, meaning multiple litters are required, or of the age at the time of scan, meaning increased variability. This is particularly important because both litter size and age are known to alter rodent brain. A recent study [7] has used theoretical simulations based on empirical data to compare the statistical powers of *in vivo* longitudinal studies and *ex vivo* cross-sectional studies for detecting group differences, with the conclusion that even when the time course is of interest, the decision is not clear-cut and there are multiple trade-offs including the number of subjects, the number of time points in longitudinal designs as well as expected effect size.

An additional concern arises from lack of clarity as to whether the volumetric measurements from *in vivo* and post mortem scans are equivalent to each other. Specifically, it is unclear whether there is tissue swelling or shrinkage caused by death and/or fixation, which would render post mortem measurements suboptimal. Previous studies have looked into the diffusion measurements from Diffusion Tensor Imaging (DTI) sequences [8]–[10], concluding that both death and fixation may alter the absolute value of the measurements, but that the relative values remain valid. DTI is a specialized type of MRI that measures the amount of water diffusivity in tissue; these findings are consistent with the known fact that the water diffusivity is reduced in fixed post mortem specimen. Another study [11] where brains were removed from the head before imaging found reduced ventricular volume in post mortem scans, presumably due to the collapsing of the ventricles in the lack of CSF pressure, and some more minor volumetric changes in other brain regions. However, this study used different populations for the *in vivo* and post mortem scans, and had the brains extracted from the skull for the post mortem scans. This excision is known to cause deformation of the brain tissue [12], making a direct comparison to *in vivo* data difficult.

In this study, we examine the brain in healthy male Wistar rats from MRI scans acquired first *in vivo* and then post mortem, at 3 age groups across adolescence (postnatal days 28 through 80). MR images of each animal were collected in 3 separate scans to compare measurement accuracy and quality between *in vivo* and post mortem scanning techniques.

Our hypotheses are: 1) Images acquired from post mortem samples will be of superior quality to those acquired from live animals, and 2) volumetric measurements of brain structures will not differ between live and post mortem scans, thus suggesting that post mortem scans are valid in studies that do not have a longitudinal design.

## Materials and Methods

### Animals

Male Wistar rats were bred and reared in our vivarium (University of North Carolina at Chapel Hill). Timed-pregnant (embryonic day 17, E17) Wistar rats were ordered from Harlan Laboratories, Inc. On the day following birth (postnatal day 1, P1), litters were culled to 10 pups. At weaning on P21, male offspring were pair-housed with a same-age non-littermate and body weight match assigned to three groups, 8 rats each group. First group were used at P28–30, second group at P42–44 and third group at P78–80. All animals remained housed as such throughout the experimental procedures until used (2 animals/cage), and maintained at 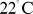 under a 12∶12 hours light/dark cycles with free access to food and water. This study was carried out in strict accordance with the recommendations in the Guide for the Care and Use of Laboratory Animals of the National Institutes of Health. All animal protocols approved by the Institutional Animal Care and Use Committees at the University of North Carolina.

### Scan Parameters and Image Acquisition

Each rat brain was scanned three times in two different sites, Biomedical Research Imaging Center at University of North Carolina at Chapel Hill, NC (as Site-1) and Center for In Vivo Microscopy at Duke University (as Site-2). All rat brains were first scanned live under anesthesia at Site-1, then sacrificed and perfused (see below for perfusion details). Then, the first post mortem scans were acquired at Site-1, and second post mortem scans were acquired at Site-2.

**Figure 1:**
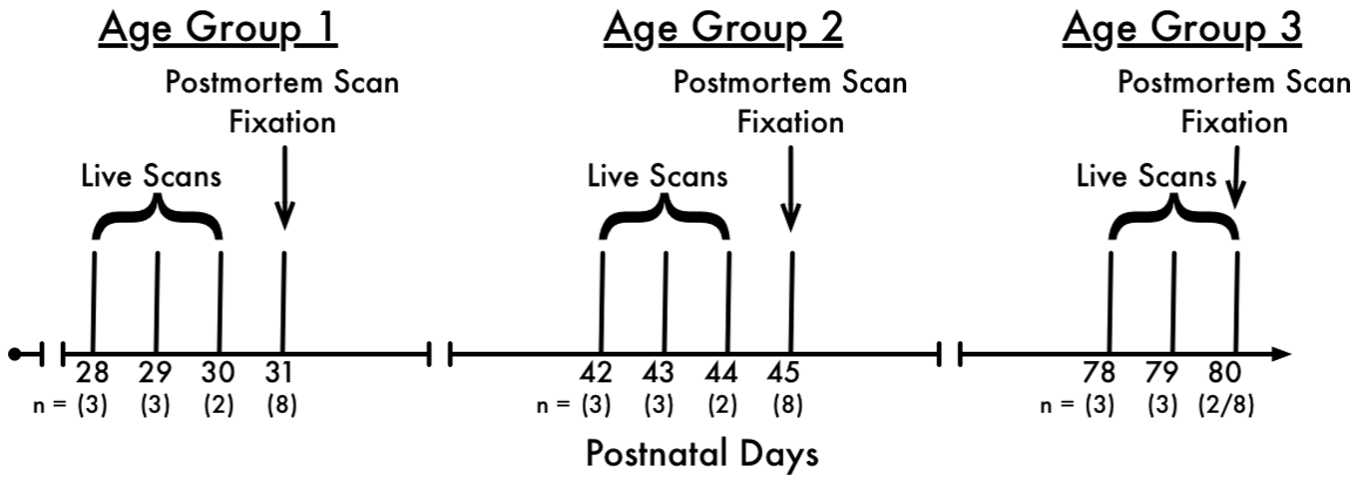
Timeline of imaging protocol.

Figure 1 depicts the sequence of events in the imaging protocol. In particular, the *in vivo* scan of the first eight rats were separately acquired at P28 (3 rats), P29 (3 rats) and P30 (2 rats) due to practical constraints on number of animals that can be scanned within a given day given the duration and preparation time for each subject. Similarly, the second group of eight rats were scanned at P42 (3 rats), P43 (2 rats) and P44 (3 rats); and the third group of eight rats at P78 (3 rats), P79 (3 rats) and P80 (2 rats). The intact skulls were removed separately at P31, P45 and P80 after *in vivo* scan and post mortem scans were acquired at Site-1. The intact skulls were then transported to Site-2 and scanned for the third and final time.

#### Perfusion protocol

Rats were anesthetized with pentobarbital sodium, and perfused transcardially with warmed saline-Prohance (1∶10 solution of Prohance in 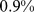 saline, 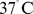) wash for 4 minutes, and then with formalin-Prohance (1∶10 solution of Prohance in 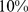 formalin) for 4 minutes at room temperature with 20 ml/min flowing rate. Heads were removed and stored in small jar of 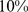 formalin (with no Prohance) for 24 hours. After 24 hours, the heads were transferred to 1∶100 solutions of Prohance in PBS and stored in 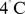 until use.

For the three age groups, the body weights averaged 104.5 g (SD = 8.2), 215.4 g (SD = 14.2), and 452.5 g (SD = 14.4) at the time of surgery, respectively.

#### In vivo scan of live rats at Site-1

Rats were scanned using a Bruker BioSpec 9.4T horizontal bore MRI system (Bruker, Billerica, MA). Images were acquired using a 4-channel phase-array surface coil with the rat in supine position. 3D MDEFT sequence was used for T1-weighted image acquisition with the following parameters: TE = 3.9 ms, TR = 4000 ms, Inversion time = 1200 ms, NEX = 1; matrix size of 256×256, and the voxel size of 0.15 mm isotropic. To improve signal-to-noise-ratio (SNR), two images were acquired immediately following each other for each animal, and these two were averaged together following rigid registration. Total scan time was two hours.

#### Post mortem scan of rats at Site-1

After sacrifice and perfusion with contrast agent, rats were rescanned at Site-1 and images were acquired using the similar 3D imaging protocol described above except for the following changes: TE = 6.7 ms, TR = 4000 ms, NEX = 4; matrix size of 320×210, and the voxel size of 0.1 mm isotropic, and acquisition time was 6 hours. To improve SNR, two images were acquired immediately following each other for each brain sample, which were averaged following rigid registration. Total imaging time was 12hours.

#### Post mortem scan of rats at site-2

Rats were scanned at Site-2 and images were acquired using a 9.4 T vertical bore Oxford Instruments magnet with shielded coils providing gradients of up to 2000 mT/m (Resonance Research, Inc., Billerica, MA), controlled by a GE EXCITE MR imaging console (GE Healthcare, Milwaukee, WI). T1-weighted MR images were acquired with a voxel size of 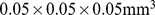, for an acquisition time of 80 minutes. Scan parameters were selected to take advantage of a technique known as active staining [13]. Active staining involves combining the PFA fixative and Prohance contrast agent together to enhance MR signal by preferentially reducing the spin lattice relaxation time (T1), while simultaneously preserving the tissue, so that conventional histology could be performed later [14]. Note that the contrast agent reverses the contrast so that water appears brighter and fat appears darker in these images. This contrast reversal does not affect any of our subsequent analyses.

### Automatic Image Segmentation

Manual outlining of regions of interest (ROI’s) in volumetric MRI scans for large numbers of animals lacks reproducibility and is tedious, error-prone and extremely time-consuming. Thus, an automatic and reliable MR image analysis pipeline for rat brain regions is crucial to longitudinally and cross-sectionally study animal models. We have used our in-house small animal image processing pipeline[15]–[17] for this purpose. To summarize, all images are first rigidly registered to an external atlas. An atlas-based probabilistic tissue classification method, followed by morphological operations, is used to skull-strip the images. Then, for each age group, we created an age-specific unbiased population atlas using a fluid-based deformable registration method [18]. This population atlas was deformably registered with the external atlas. The ROI segmentations defined on the external atlas were mapped first to the population atlas and then to the individual subjects.

We used a previously built in-house adult rat brain atlas for the automatic processing pipeline outlined above. This atlas consists of 6 control male Wistar rats imaged post mortem at postnatal day 72 and it has been manually segmented into 22 ROI’s by our anatomical expert.

Our results from a previous study [17] have shown that the atlas-based regional analysis of volume is highly stable and structural segmentation indistinguishable from expert rater for all major structures. Additionally, at every step of the automatic processing pipeline, the intermediate results were visually inspected for quality control, and manual adjustments were performed when necessary to ensure accurate regional segmentation. The resulting segmentations were used to compute regional volume measurements.

## Results

The results from this study will be divided into two sections that address our two hypothesis: First, we will investigate whether the measurements from post mortem scans are consistent with the measurements from *in vivo* scans. Next, we will compare the image quality between the two methods to evaluate whether post mortem images are superior in quality to *in vivo* images. Animal MRI brain volumes provide an excellent opportunity for translational studies. These studies assess rat brain MRI volumes in both live and post mortem conditions. Post mortem MRI greatly reduces some complications of rat MR imaging. However, the conclusions of the studies presented here are applicable only for our methodology of fixation and whole head scans and cannot be generalized to all post mortem procedures.

### Measurement Accuracy

The main concern with using post mortem specimen for scanning purposes is that the tissue may be significantly altered compared to *in vivo* scans. Potential reasons for such a change include the fixation process as well as the death. Clearly, if the post mortem measurements are different from the *in vivo* measurements, these concerns would be proven valid, and any studies considering post mortem scanning would need to be handled with extreme care. However, our main hypothesis is that there will be no significant differences in the volumetric measurements between *in vivo* and post mortem scans, neither for individual ROI’s nor for the total brain volume.

#### Total brain volume

As a first investigation into the affects of fixation into MR-based analysis, we evaluated the total brain volume (including brainstem, cerebellum and ventricles) measurements across the different scan types for all three age groups. The analysis showed no major changes in total brain volume between *in vivo* and post mortem measurements; while there were some observed differences between groups, careful inspection of the data showed these changes can be attributed to the amount of brainstem included in the scan (due to guillotine positioning at the time of sacrifice, as well as later sample positioning in the scanner).

**Figure 2:**
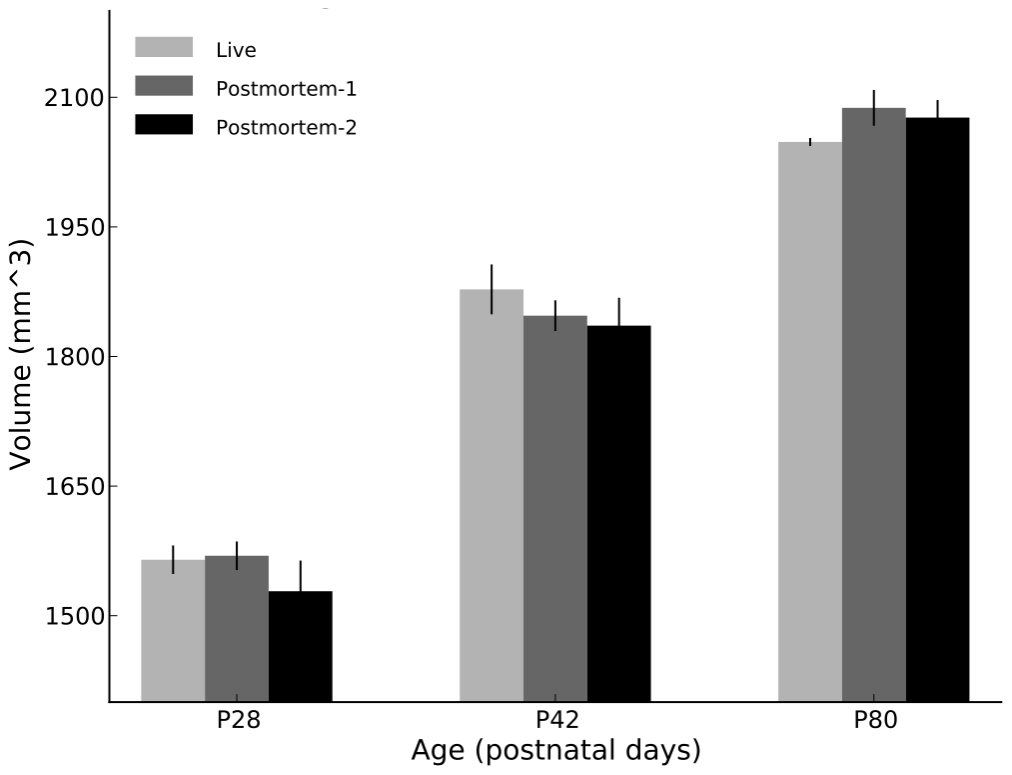
Total brain volume for each age group/scan type.

The average total brain volume as a function of scan are shown in Figure 2. 1-way ANOVA tests showed no significant effects by scan type (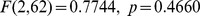), and significant differences between each age group (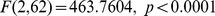), as hypothesized. Pairwise comparisons showed that the 3 age groups are statistically different from each other, as expected. 2-way ANOVA tests revealed no further differences: 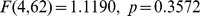.

#### Volume-weight correlations

**Figure 3:**
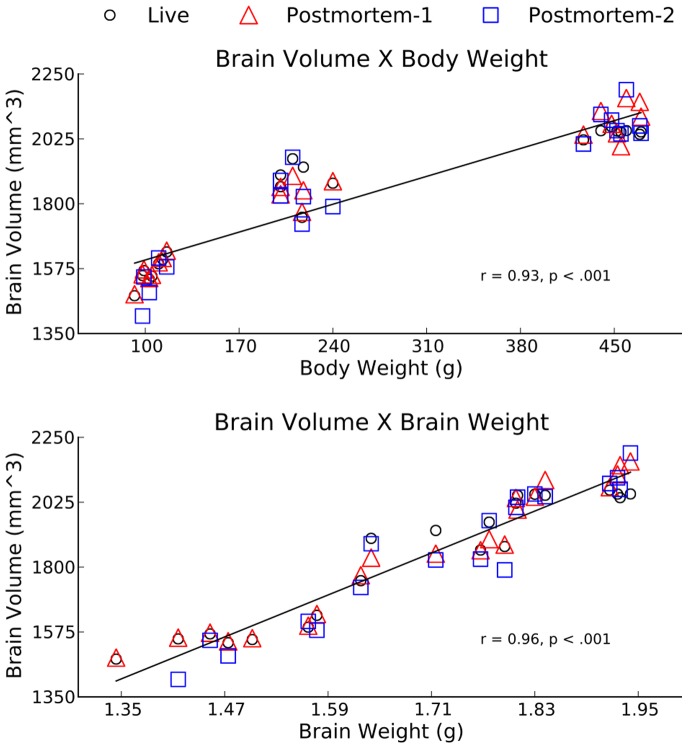
Pearson’s correlation between brain volume, brain weight and body weight.

To establish a higher level of confidence in our MRI-based measurements, we have investigated the correlation between MRI-based volume measurements and weight. Pearson’s correlations between whole brain volume, body weight, and brain weight are shown in Figure 3. Brain volume was very strongly correlated with body weight (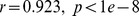 for Live, 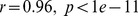 for Postmortem-1, 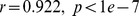 for Postmortem-2) and even more so with brain weight (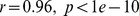 for Live, 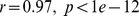 for Postmortem-1 and 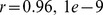 for Postmortem-2). These very strong correlations indicate that the MRI-based volume measurements are indeed accurately representative of the anatomy. In particular, note that both post mortem scans are just as strongly correlated as the *in vivo* scan, suggesting no loss of accuracy.

#### Volume of individual ROI’s

**Figure 4:**
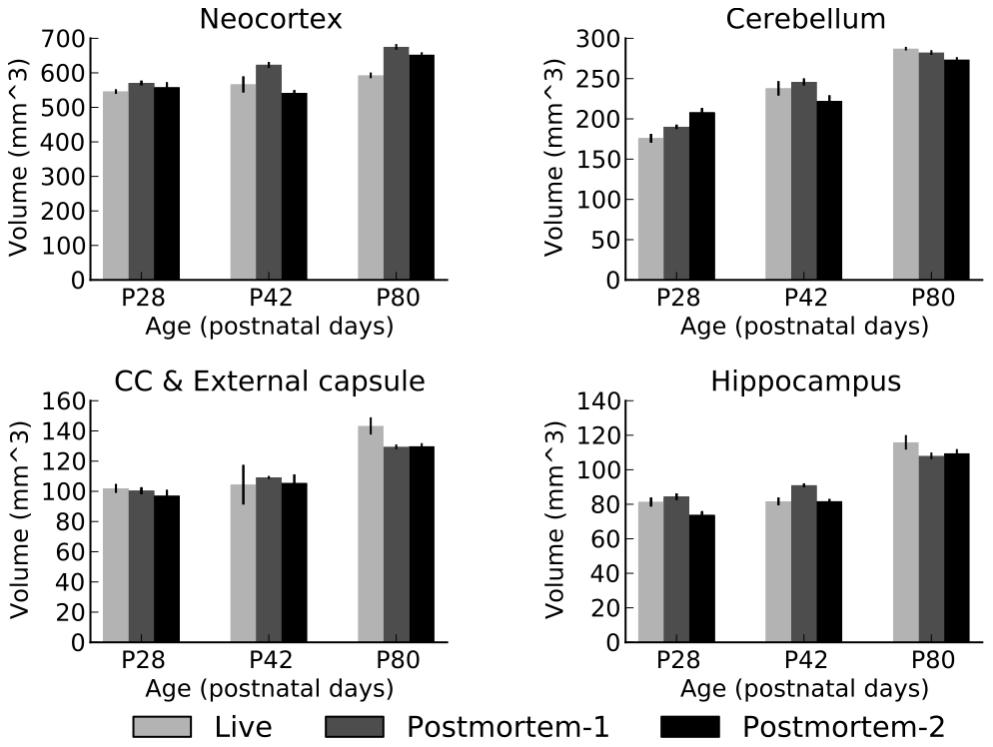
Volume of selected regions as a function of age and scan type.

For a finer-level investigation into the effects of fixation on structural volume as measured by MRI, we evaluated the volume measurements from 22 individual ROI’s across the 3 age groups and the 3 scan types. The results from 4 selected structures are presented in Figure 4. These 4, the neocortex, hippocampus, corpus callosum and cerebellum were chosen to be representative of both white and gray matter structures. This is an important concern since previous studies such as [12] demonstrated that the various brain regions may be affected differentially by death and fixation, especially if the brain is extracted from the skull prior to imaging. The structures charted on Figure 4 are all consistent (within each age group) with each other (up to noise/measurement error), further supporting our hypothesis that the post mortem scans are just as reliable a source of information as *in vivo* scans.

The summary statistics for the volume measurements for each individual anatomical structure examined are tabulated as a function of age and scan type in Table 1. Note that the rows are ordered by decreasing size. Typically, the confidence in the segmentation also decreases with size, since it becomes increasingly difficult to delineate boundaries of small regions unless they present a very strong MR contrast. For example, the VTA spans about 15–20 voxels in the Live scans, which is smaller than a 3×3×3 cube. Even in the high-resolution Postmortem-2 scans, these smaller regions are challenging to segment consistently, whether automatically or by human experts. In comparison, the neocortex spans more than 150,000 voxels on average, and is relatively easy to segment consistently.

**Table 1:** Descriptive statistics for volumetric measurements for each age group and scan type.

	P28			P42			P80		
	Live	PM-1	PM-2	Live	PM-1	PM-2	Live	PM-1	PM-2
Neocortex	545.6 (14.9)	570.7 (18.6)	557.7 (32.5)	566.4 (46.4)	623.3 (20.5)	541.7 (20.9)	592.9 (22.1)	675.3 (23.8)	652.6 (20.7)
Cerebellum	175.9 (14.1)	190.1 (7.4)	207.9 (12.4)	237.9 (17.7)	245.9 (10.4)	222.2 (17.3)	287.2 (6.1)	282.4 (8.4)	273.6 (8.9)
Brain Stem	106.1 (6.6)	80.6 (6.2)	134.7 (12.2)	155 (8.4)	161.1 (3.1)	147.4 (13.1)	191.1 (7.6)	179.7 (8.2)	209 (8.2)
CC & External capsule	102 (7.4)	100.4 (6.5)	97 (8.7)	104.4 (26.2)	109.2 (2.1)	105.5 (14)	143.3 (15.1)	129.5 (4.4)	129.8 (5.9)
Olfactory Bulb	70.7 (8.1)	79 (5.6)	97.3 (6.1)	89.7 (9.2)	100.9 (2.3)	85 (5.7)	123 (5.7)	119.7 (13.4)	119.6 (3.7)
Basal Forebrain	88.4 (3.2)	83.3 (2.7)	78.9 (5)	102 (15.4)	98.4 (3.2)	93.1 (5.4)	102.5 (8.1)	109.5 (2.4)	111.1 (3)
Hippocampus	81.3 (6.7)	84.5 (5.3)	73.8 (5)	81.7 (4.5)	91 (2.7)	81.7 (3.2)	115.9 (11.1)	108.1 (5.5)	109.5 (7)
Dorsal Striatum	81.1 (8.9)	68.5 (3.8)	62.2 (1.6)	81.4 (8.8)	72.6 (1.3)	74.6 (3.3)	100.1 (8.9)	89.3 (3.8)	88.5 (4.6)
Rest of Midbrain	62.4 (3.5)	78.2 (5)	54.8 (5.7)	72.1 (7.9)	78.6 (2)	78.5 (5.1)	97.6 (2.2)	91 (2.5)	84 (3.1)
Hypothalamus	51.6 (4.6)	50 (1.1)	39 (2.2)	59.8 (4.7)	59 (2)	49.4 (3.8)	52.8 (2.7)	62.4 (3.1)	60.2 (3.6)
Thalamus	50.7 (6.2)	39.7 (1.9)	36.3 (1.8)	62.4 (8.1)	47.9 (1.4)	51.7 (2.7)	63.1 (4)	58.8 (5.4)	55.9 (3.6)
Inferior colliculi	19 (1.7)	23.8 (3.3)	21.7 (2.1)	22.4 (6.5)	27.5 (1.1)	22.5 (3.1)	30.2 (6.5)	31.2 (1.1)	26.3 (1.6)
Amygdala	17.9 (3.3)	19.6 (1.1)	16.8 (0.3)	16.3 (3.8)	20.2 (1.1)	15.2 (0.6)	16.6 (2)	22.1 (0.9)	22.6 (0.7)
Fimbria	14.5 (1)	11.9 (0.9)	12.7 (0.8)	17 (5.5)	13.9 (0.6)	15.9 (0.9)	23 (4.2)	18.4 (2.2)	20.5 (1.9)
Central Gray	12.6 (2.5)	16.2 (1.9)	9.8 (0.8)	13.1 (3)	15.7 (0.4)	15.4 (1.1)	21.4 (1.1)	18.2 (0.9)	17.3 (0.6)
Internal capsule	10.3 (2.9)	9 (0.4)	8.9 (1.6)	11.5 (3.1)	9.6 (0.3)	9.7 (3.2)	11.5 (2.9)	11.1 (0.5)	12.9 (0.7)
Ventricle	9.9 (1.1)	7.9 (0.4)	4.6 (0.5)	12.5 (2.4)	10.6 (0.4)	10.3 (0.7)	17.3 (1.1)	12.9 (0.9)	10.6 (1.1)
AC	5.1 (1)	4.9 (0.2)	4.5 (0.6)	4.7 (0.7)	5.9 (0.2)	5.4 (0.5)	6.7 (0.6)	6.8 (0.2)	6.9 (0.3)
Superior Colliculi	5.4 (0.4)	8 (0.8)	5.5 (1.5)	5.1 (0.5)	6 (0.2)	6.2 (0.8)	8.1 (0.5)	7.6 (0.4)	6.1 (0.5)
Substantia Nigra	2.6 (0.5)	3.3 (0.2)	2.2 (0.5)	3.4 (0.3)	3.5 (0.3)	3.4 (0.3)	4.2 (0.6)	4 (0.4)	4 (0.3)
Fornix	1.6 (0.3)	1.4 (0.2)	1.7 (0.3)	1.6 (1.8)	1.8 (0.2)	2.9 (0.5)	3.4 (0.9)	2.8 (0.5)	2.6 (0.4)
VTA	0.09 (0.03)	0.3 (0.05)	0.46 (0.17)	0.05 (0.03)	0.36 (0.03)	0.59 (0.25)	0.15 (0.05)	0.39 (0.06)	0.61 (0.06)
Total Brain	1564.8 (46.8)	1569.4 (46.7)	1528.3 (79.4)	1877.7 (70.7)	1847.3 (43.5)	1835.7 (79.2)	2048.3 (13.1)	2087.8 (58.5)	2076.5 (57.4)

Given the consistency between the volumetric measurements in the *in vivo* and post mortem scans, death and fixation do not appear to have a systematic affect on the volumes of individual brain regions or the total brain volume. Note that previous studies [12] have shown an increase in volume when the brain is extracted from the skull and soaked in fixative for histology purposes. However, in this study, the brains were perfused with fixative within the skull and not extracted, so the volumetric measurements are not affected by the inhomogeneous swelling that occurs in extracted brains. Furthermore, the MRI-based measurements are not subject to the distortions that commonly plague the fragile brain tissues when they are extracted from the skull and undergo sectioning.

**Figure 5:**
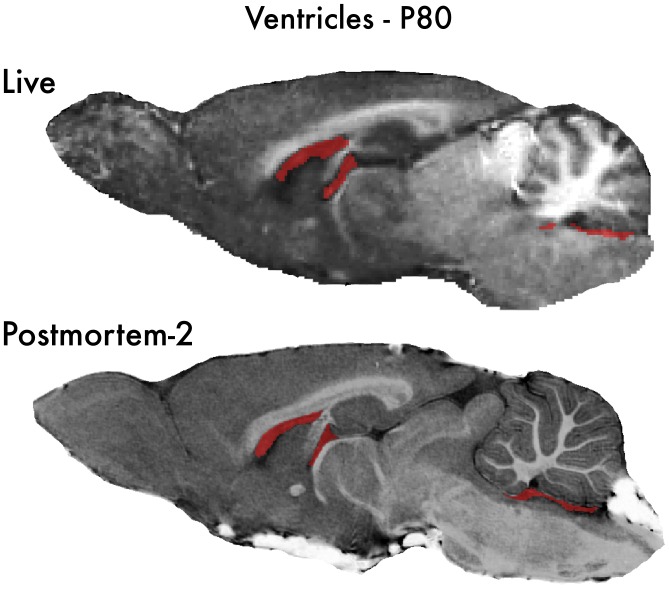
Ventricles in Live and Postmortem brains. Sagittal sections that highlight the lateral ventricles are shown for a live and Postmortem-2 brain scan of a P80 young adult animal. Ventricular spaces are highlighted in red. Note the post mortem image shows clear brain regional morphology, whereas the live image shows discretization artifacts in the highlights. These differences in image quality would be expected to improve segmentation accuracy and quantification of ventricles similar to other brain regions. However, the ventricles are fluid-filled spaces that are more likely to be altered by post mortem procedures. Although our ventricular volumes were not statistically different in live vs post mortem comparisons, at each age studied there is an apparent trend of reduced volumes from live to Postmortem-1 and Postmortem-2, which suggests post mortem ventricular values should be interpreted with particular caution compared to the other brain regions of interest.

Ventricular volumes are commonly studied using human MRI due to the easy distinction of the fluid filled ventricles from the surrounding brain tissues. Ventricle volumes are often used to assess atrophy in human studies. We found that our post mortem scan ventricular volumes were not statistically different from in vivo values at P28, P42 or P80 (Table 1, Figure 5). Although there is considerable variation, post mortem images containing contrast agent should more easily define the ventricular borders (Fig. 5). However, there is a trend for decreasing ventricular volumes at each age group from live to Postmortem-1 to Postmortem-2 measures (Table 1). Postmortem-2 scans were on average 2 months after the *in vivo* scans. Better post mortem image resolution would be expected to improve ventricle measures, however, the trend in reduced post-mortem ventricular mean size suggests ventricular volumes should be interpreted with caution since they are spaces more difficult to preserve with fixation. Although our studies find post mortem MRI brain volume measures using our fixation techniques are able to replicate *in vivo* brain regional volumes, ventricle space values appear most at risk for post mortem disruption.

### Image Quality

The volumetric data showed no significant differences in measurements between *in vivo* and post mortem scans, thus establishing the post mortem scan as a valid measurement tool. However, to show that it is in fact *preferable* to acquire post mortem scans whenever the experimental design allows it, it is necessary to compare the image quality from the different methods. For this purpose, we present both visual assessments and quantitative data showing improved image quality in post mortem scans.

#### Visual quality assessment

**Figure 6:**
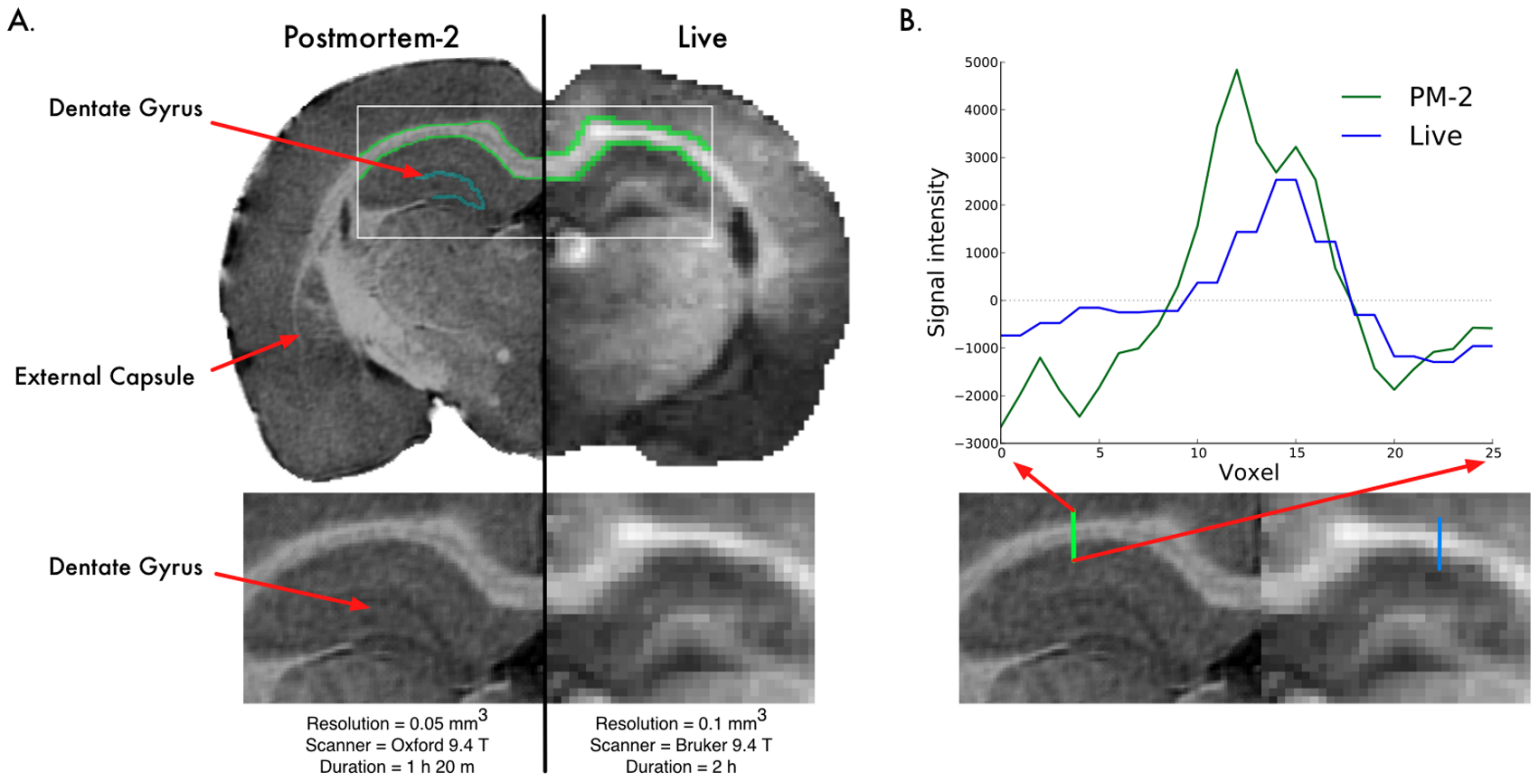
Image quality. (A) visual assessment. (B) intensity profile across the corpus callosum.

We start by visual inspection of the images. Figure 6 depicts a comparison between the *in vivo* scan and the high resolution Postmortem-2 scan of the same animal. The increased resolution in post mortem scans clearly allows for a much sharper contrast between various structures. This enables the identification of smaller structures, e.g. the dentate gyrus or thin white matter structures such as the inferior portion of the external capsule. These structures could be readily analyzed automatically, just like any of the ROI’s presented in Section 0. In contrast, *in vivo* scanning is limited in resolution and makes it difficult to determine the boundaries of such regions even for a human expert. Additionally, the blurring of boundaries caused by lower resolution decreases the confidence with which the exact location of structure boundaries can be located even for larger structures such as the corpus callosum.

In order to provide concrete evidence regarding the increased contrast in the post mortem scan, we evaluated the intensity profile in the images across the corpus callosum (Figure 6, right). The intensity values from the two scans were extracted along a straight vertical line across the corpus callosum. The values were centered and plotted in Figure 6 (note that the Postmortem-2 values were inverted in order to make them comparable, due to the presence of the Prohance which reverses the tissue contrast). The Postmortem-2 image showed a very sharp intensity peak as the profile crossed from the neocortex (gray matter) to the corpus callosum (white matter), and a very sharp decline as it crossed back to gray matter (hippocampus). In comparison, the intensity changes were quite muted in the Live scan, which captures the visual assessment of a ‘less sharp’ image. This is largely due to the blurring in the Live image caused by partial voluming effects as well as potential motion artifacts; additionally, the Postmortem-2 image benefits from the Prohance agent which further improves contrast.

#### Quality quantification via SNR measurements

To quantify these visually evident quality differences across the scan types, we calculated the signal-to-noise ratio (SNR) [19]. To calculate the SNR, a ‘foreground’ and a ‘background’ region were chosen for each scan. The background region was manually chosen as a cube shaped region outside the brain image, away from any systematic intensity artifacts, with an edge length equal to 20 voxels. The foreground region was chosen as the entire brain area on the midsagittal slice by using the skull-strip mask previously computed, eroded isotropically by 5 voxels to ensure containment well within brain boundaries. The SNR of the image was then calculated as the ratio of the average foreground intensity to the standard deviation of the background intensity.

**Figure 7:**
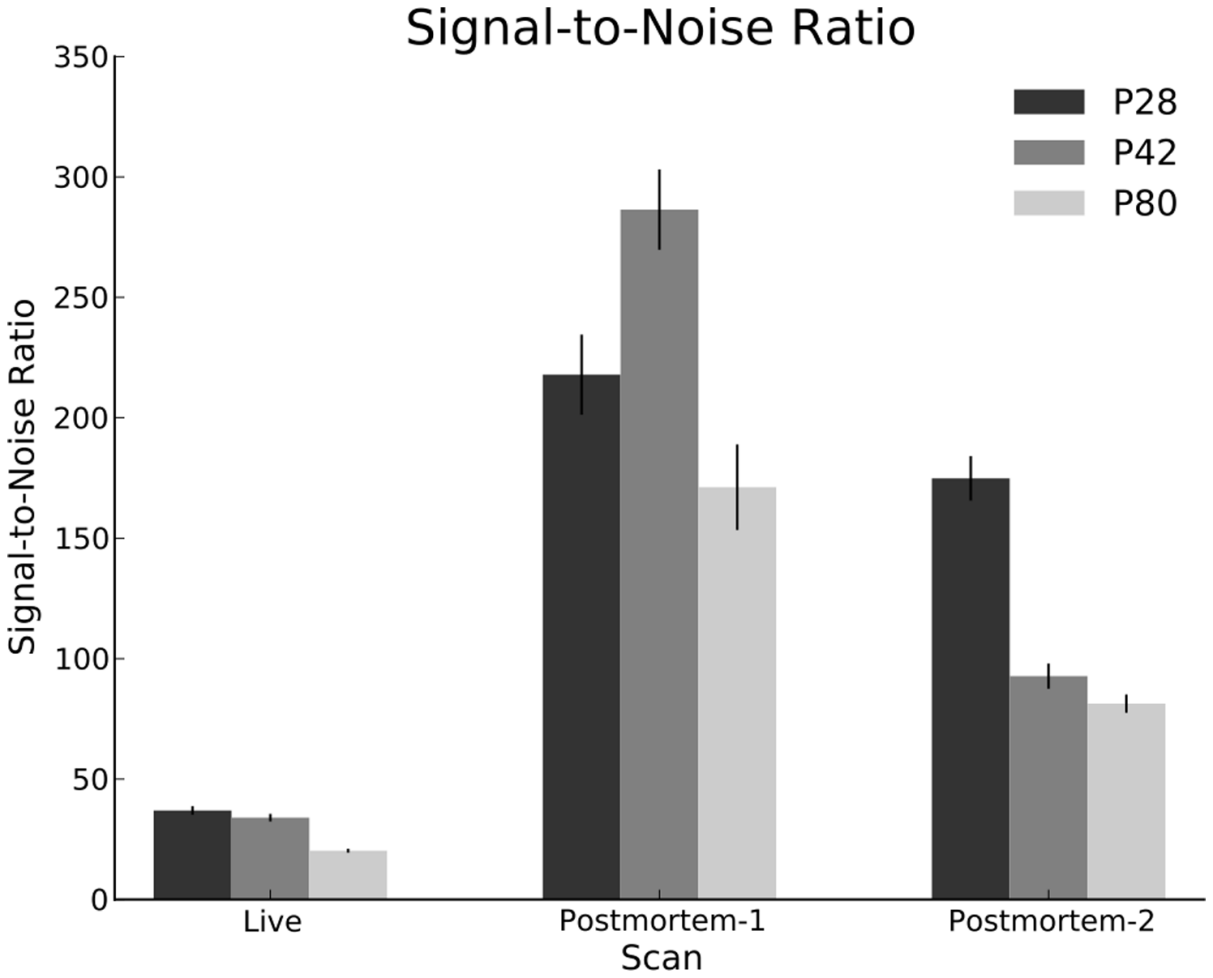
Signal-to-noise ratio.

The average SNR values as a function of age and scan are shown in Figure 7. The *in vivo* scans have much lower SNR as compared to the two post mortem scans, which further supports the reduced image quality argument. 1-way and 2-way ANOVA analysis confirms these observations: Age 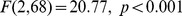; Scan 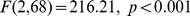; Age X Scan 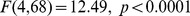.

It should be noted that the SNR values can vary considerably by age within a scan type. Several factors contribute to this phenomenon, such as the specimen size, resolution and matrix size. The specimen size is considerably different between the age groups, as evidenced by the total brain volume analysis above. The relative size of the specimen to the matrix size affects the SNR. A smaller specimen can load the RF coil less, yielding a higher SNR. Additionally, the SNR is directly proportional to the volume of each voxel, which is determined by the image resolution. Furthermore, the contrast agent may perfuse differently into the older, larger brains. Despite all these interacting factors, all the post mortem scans were found to have significantly higher SNR than all the *in vivo* scans, regardless of age, acquisition protocol and scanner type.

As an additional indicator of image quality, we have compared the standard deviation in the various ROI’s listed in Table 1. A reduced standard deviation indicates lower measurement noise. For this comparison, we used a basic paired t-test between the standard deviations in the volume measurements in the Live scans and the Postmortem-1 scans (Live vs Postmortem-2 comparison showed similar results). We found the standard deviation to be significantly reduced in post mortem scans (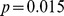, 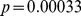, and 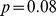 for the three age groups). The two main causes of the increased variability in the live scans are the age variation in the live scans due to the staggered timeline imposed by scan logistics, as well as the noise and measurement uncertainty in the *in vivo* scan. These findings further support the argument for using post mortem scans to improve image quality and the reliability of the image processing methods, ultimately yielding increased statistical sensitivity.

## Discussion

This comparison of *in vivo* and post mortem scans confirms our 2 hypothesis. First, post mortem scans are shown to be a valid and valuable option in studies that do not require a longitudinal design. Brain regional volumes found in live scans do not differ from post mortem scans of brains perfused with fixative in situ and imaged within the head. In particular, the segmentation and volume measurement of the 22 ROI’s reveals that Live measurements often fall between the two post mortem measurements, suggesting that the variations are within the noise rather than being a biological phenomenon causing a bias. The post mortem volumetric measurements are also found to have extremely strong correlations with body weight and brain weight, further supporting the validity of these measurements. While there are some regions where either the *in vivo* or the post mortem scans seem to have larger volume, similar to what’s observed in [11], there are regions showing trends in both directions (in vivo 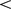 post mortem or post mortem 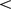 in vivo); additionally, these differences do not reach statistical significance when we correct for multiple comparisons; therefore, we conclude that these differences are likely to be due to methodological variability, such as segmentation noise. The tight match between total brain volumes of the individiual animals as measured via different scan types (Figure 3) further supports this argument.

It should be noted that in human brain studies, longitudinal design has great value due to the variation of age and weight between subjects, as well as dramatically increased variability in brain structure between individuals. In rodent studies, researchers can control variables such as age and weight and litter size, reducing the necessity for longitudinal study designs. Additionally, anesthetics and alcohol activate cortisol stress responses, as does restraint [20]. Repeated longitudinal MRI requires repeated anesthetic and restraint that would be expected to confound longitudinal studies of animals, particularly in alcohol-related experiments.

Our findings of post mortem brain regional volumes reflecting *in vivo* volumes supports the validity of post mortem studies of volume. Experimental design of developmental changes in rat brain during adolescence is difficult using live scans. For example, at each of the age groups studied in this manuscript, 3 days of scanning multiple brains per day were necessary to scan the 8 brains per group. Rats continue to grow with age, increasing body weight through adolescence and well into adulthood. In our study from postnatal day 28 to postnatal day 80, rats grew from about 100 g to about 450 g. The increase in body weight correlated with increased brain volume, although brain volume increased by about 33%, whereas body weight increased by 450%. The growth of brain with increased age confounds longitudinal studies since animals are continuously growing. Further, live scans are confounded by differences in animal age during scanning. Although we were able to do live scans of all 8 animals within 3 days, spacing of live scans across a longer period of time would be expected to result in considerable brain volume changes due to the different ages at which subjects were scanned. For example, in the 14 days between P28 and P42 brains of animals grew from a mean of 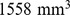 to 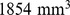, an 18% increase in controls due to aging that would confound comparisons of studies on genetic, strain, disease models or other factors that might alter brain structure. We devoted considerable effort to control litter size and age to obtain homogeneous groups of similar age. It would be very difficult to have multiple litters staggered to have animals at the same age for live scanning across development. Although longitudinal studies would appear to resolve this issue, restraint, anesthesia and the scanning process are stressful to the animal impacting brain growth and reducing the validity of live scans. In contrast, animal sacrifice, brain fixation and post mortem scanning allows animals to be nearly identical in age and body weight when scanned, reducing variability and the complexity of experimental design. Thus, our studies finding nearly identical brain volumes for live and post mortem scans across adolescent brain development provide key information validating our post mortem approach which greatly simplifies experimental design and lays the foundation for additional studies on factors that could impact brain development. In particular, Postmortem-2 scans were acquired 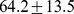 days after the in vivo scan, and we showed that there are no significant differences between these measurements. This illustrates the flexibility post mortem imaging offers in terms of experimental design in contrast to the live imaging paradigm, since we have found remarkably similar measurements between these scans and both the live scans and the Postmortem-1 scans that were acquired earlier.

We found large changes in brain volumes across age that correlate with body weight. Our absolute brain regional volume values change dramatically with age. Normalization of brain regional volumes for body size or intracranial volume might reduce variation, improving accuracy. However, our studies provide methods that allow tight absolute volume measurements due to the ability to fix the brain of an entire group of animals at a specific time point while preserving absolute volume values. Thus, our studies presented here provide methods allowing experimental design to capture specific ages and time points following treatments or disease onset.

Additionally, the image quality is shown to be dramatically improved in post mortem studies. We believe several reasons contribute to this improvement: lack of motion/pulsation artifacts; the contrast agent used during the perfusion and increased scan time which allows for increased resolution and signal-to-noise ratio. Note that our perfusion protocol, which uses the active staining technique, is rather unique and we believe it strongly contributes to our ability to reproduce *in vivo* measurements using post mortem scans. In particular, the protocol includes keeping the perfusion solution warm to increase the likelihood of the contrast agent to be evenly distributed. This also evenly distributes the fixative which cross links molecules making the brain hard and stabilizing its structure.

The signficance of the improved image quality is twofold. First, better images result in higher confidence in the segmentation of subcortical structures. Even relatively large structures such as the corpus callosum or external capsule are hard to segment precisely given the blurry boundaries in the *in vivo* images. Clearly, segmentations with higher confidence levels will lead to more precise measurements and ultimately, improved statistical power in studies. Second, these higher resolution images will allow researchers to segment finer structures that may not be visible in *in vivo* images. The dentate gyrus, illustrated in Figure 6, is one such structure. This will enable the researchers to investigate smaller structures individually, rather than aggragating them in large combined structures. This may lead to the detection of subtle differences that would otherwise be averaged away within the large combined region.

As an additional aim, we have compared post mortem scans at two different MRI facilities with different acquisition protocols. An additional difference was time elapsed between perfusion and scan (Postmortem-1 scans were always acquired before Postmortem-2 scans). Our results show that these factors do not significantly contribute to the volumetric measurements. This further establishes the post mortem MRI as a valid and robust tool for volumetric analysis of the rodent brain.

### Conclusions

We presented a study comparing *in vivo* and post mortem scans in healthy male Wistar rats throughout adolescence (postnatal days 28 through 80). The animals were first scanned *in vivo*, immediately followed by perfusion. The specimen were then scanned post mortem at two different MRI facilities with different magnets and acquisition protocols. We have evaluated the accuracy of the volumetric measurements collected from 22 individual ROI’s in the brain as well as whole brain volume. The results showed that there were no significant changes in the measurements between the three scan types. We also compared the image quality from the different scan types, and found that the post mortem scans were dramatically superior in quality regardless of age and acquisition protocol.

These findings establish post mortem MR imaging as a valid method for reliable volumetric measurements from rodent brains. The post mortem scans are also shown to be of superior quality. However, post mortem scanning clearly does not allow for longitudinal studies. Ultimately, the choice between *in vivo* and post mortem scans need to be made based on the specifics of the study design.
